# Panton-Valentine Leukocidin–producing *Staphylococcus aureus*

**DOI:** 10.3201/eid1211.060726

**Published:** 2006-11

**Authors:** Amos Adler, Violeta Temper, Colin S. Block, Nitsa Abramson, Allon E. Moses

**Affiliations:** * Hadassah-Hebrew University Medical Center, Jerusalem, Israel;; † Ministry of Health, Jerusalem, Israel

**Keywords:** Staphylococcus aureus, Panton-Valentine leukocidin, skin infections, interfamilial outbreak, letter

**To the Editor:** Panton-Valentine leukocidin (PVL) is a cytotoxin produced by *Staphylococcus aureus* that causes leukocyte destruction and tissue necrosis (*1*). Although produced by <5% of *S. aureus* strains, the toxin is detected in large percentages of isolates that cause necrotic skin lesions and severe necrotizing pneumonia (*2*). Although commonly associated with community-acquired methicillin-resistant *S. aureus* (CA-MRSA) (*3*), several outbreaks due to methicillin-susceptible *S. aureus* (MSSA) have also been reported (*4**–**6*). We describe an outbreak of cutaneous infections caused by PVL-producing MSSA that affected 6 of 11 members of 2 related families.

During a period of 6 months, a cluster of *S. aureus* skin and soft tissue infections occurred in 2 families in Jerusalem, Israel, that were related through the mothers, who are sisters. The event started with the 4-year-old boy of family A, who had 5 episodes of skin infections, including 2 episodes of perianal abscesses that required drainage and hospitalization. Culture of pus grew MSSA that was resistant to erythromycin and clindamycin. Subsequently, recurrent abscesses and cellulitis developed in the boy's father's legs, and his mother had severe periorbital cellulitis that required hospitalization and surgical drainage. Approximately 1 month later, a 9-year-old boy in family B had severe cellulitis and abscess around his knee that required hospitalization and surgical drainage. Subsequently, infections developed in 2 more children in family B: 1 had a finger pulp-space infection and the other cellulitis of the lower abdomen. All pus cultures grew *S. aureus* with identical susceptibility patterns. The cases are summarized in the Table.

**Table:** Clinical and microbiologic data of the outbreak*

No.	Family	Sex/age (y)	Clinical manifestations	Pus cultures	Nasal cultures	PFGE pattern/PVL
1	A	F/33	Periorbital cellulitis	MSSA, Ery/clin- R	MSSA, Ery/clin- R	I/P
2	A	M/36	Leg cellulitis/abscess	None	MSSA, Ery/clin- R	I/P
3	A	M/4	Perianal abscess	MSSA, Ery/clin- R	MSSA, Ery/clin- R	I/P
4	A	F/2	None		N	
5	A	F/1	None		N	
6	B	F/37	None		MSSA, Ery/clin-R	I/P
7	B	M/38	None		MSSA, Ery/clin-R	I/P
8	B	M/9	Knee cellulitis/abscess	MSSA, Ery/clin- R	MSSA, Ery/clin- R	I/P
9	B	M/4	Finger-pulp infection	None	MSSA, Ery/clin- S	D/N
10	B	F/3	Lower abdomen cellulitis	None	N	
11	B	F/3	None		N	

*PFGE, pulsed-field gel electrophoresis; PVL, Panton-Valentine leukocidin; F, female; MSSA, methicillin-sensitive *Staphylococcus aureus*; Ery, erythromycin; clin, clindamycin; R, resistant; I, identical strain; P, positive; M, male; N, negative; S, sensitive; D, different strain.

Following these events, the families consulted the infectious diseases clinic at the Hadassah-Hebrew University Medical Center in Jerusalem. Since the clinical isolates were not available, nasal cultures were obtained from all family members. *S. aureus* was isolated from all the affected members of family A and from the parents and the 2 boys in family B. All 7 isolates were subjected to pulsed-field gel electrophoresis (PFGE) after digestion with *Sma*I. All except 1 had identical band patterns and the same antimicrobial drug susceptibilities as the clinical isolates. The presence of PVL genes was examined by PCR as previously described (*2*) and was detected only in the isolates with identical PFGE patterns. The families were advised to apply mupirocin nasal ointment twice a day for 5 days and to bathe with 4% chlorhexidine scrub for 1 week (*7*). At 7 months of follow-up, no new cases of skin infection had occurred in either family. An epidemiologic investigation was undertaken by the local department of health to determine if 3 kindergartens and 2 schools attended by the 7 children had an increased incidence of staphylococcal skin disease. No evidence of unusual disease was found.

We describe here the first confirmed cases of PVL-producing *S. aureus* infections in Israel. Maier et al (*8*) recently described 2 cases of similar infections that occurred in German tourists after visiting the Dead Sea area, but since these infections were caused by MRSA, it is probable that the isolates were genetically distinct from the strain described here. In addition, to the best of our knowledge this is the first description of transmission of PVL-producing MSSA between related families. Previous reports described community-related outbreaks that occurred within families (*6**,**8**,**9*), between schoolmates (*4*), and between football team players (*10*). The exact route of transmission was not identified in some of these cases but it was presumed to have been close contact leading to skin (*10*) or nasal (*4*) colonization and subsequent active infection. In our report, the PVL-producing *S. aureus* clone was detected in nasal cultures in 6 of the 11 members of the 2 families. In this niche, it was able to persist and cause a series of infections in a relatively large number of family members. Even though the *S. aureus* isolated from active lesions were not available for testing, the recovery of identical PVL-positive organisms from nasal cultures strongly suggests the presence of a pathogenic clone that probably caused the recurrent infections in the 6 affected family members. Our investigation highlights the high transmissibility of this PVL-producing *S. aureus* clone, its high attack rate, and its virulence. The intervention in this outbreak might have prevented not only subsequent recurrences of cutaneous infections but also further spread of this clone and the manifestation of even more serious infections such as necrotizing pneumonia. Increasing awareness among community-based healthcare providers of PVL-producing *S. aureus* infections is important to facilitate rapid and adequate response in similar clinical events in the future.
